# The Relative Contribution of Paracine Effect versus Direct Differentiation on Adipose-Derived Stem Cell Transplantation Mediated Cardiac Repair

**DOI:** 10.1371/journal.pone.0059020

**Published:** 2013-03-19

**Authors:** Dezhong Yang, Wei Wang, Liangpeng Li, Yulan Peng, Peng Chen, Haiyun Huang, Yanli Guo, Xuewei Xia, Yuanyuan Wang, Hongyong Wang, Wei Eric Wang, Chunyu Zeng

**Affiliations:** 1 Department of Cardiology, Daping Hospital, Third Military Medical University, Chongqing, China; 2 Department of Ultrasonography, Southwest Hospital, Third Military Medical University, Chongqing, China; 3 Department of Plastic Surgery, Daping Hospital, Third Military Medical University, Chongqing, China; Northwestern University, United States of America

## Abstract

**Background:**

Recent studies have demonstrated that transplantation of adipose-derived stem cell (ADSC) can improve cardiac function in animal models of myocardial infarction (MI). However, the mechanisms underlying the beneficial effect are not fully understood. In this study, we characterized the paracrine effect of transplanted ADSC and investigated its relative importance versus direct differentiation in ADSC transplantation mediated cardiac repair.

**Methodology/Principal Findings:**

MI was experimentally induced in mice by ligation of the left anterior descending coronary artery. Either human ADSC, conditioned medium (CM) collected from the same amount of ADSC or control medium was injected into the peri-infarct region immediately after MI. Compared with the control group, both ADSC and ADSC-CM significantly reduced myocardial infarct size and improved cardiac function. The therapeutic efficacy of ADSC was moderately superior to ADSC-CM. ADSC-CM significantly reduced cardiomyocyte apoptosis in the infarct border zone, to a similar degree with ADSC treatment. ADSC enhanced angiogenesis in the infarct border zone, but to a stronger degree than that seen in the ADSC-CM treatment. ADSC was able to differentiate to endothelial cell and smooth muscle cell in post-MI heart; these ADSC-derived vascular cells amount to about 9% of the enhanced angiogenesis. No cardiomyocyte differentiated from ADSC was found.

**Conclusions:**

ADSC-CM is sufficient to improve cardiac function of infarcted hearts. The therapeutic function of ADSC transplantation is mainly induced by paracrine-mediated cardioprotection and angiogenesis, while ADSC differentiation contributes a minor benefit by being involved in angiogenesis.

*Highlights* 1 ADSC-CM is sufficient to exert a therapeutic potential. 2. ADSC was able to differentiate to vascular cells but not cardiomyocyte. 3. ADSC derived vascular cells amount to about 9% of the enhanced angiogenesis. 4. Paracrine effect is the major mechanism of ADSC therapeutic function for MI.

## **Introduction**

Cardiovascular disease is a leading cause of human death worldwide [1]. Acute and chronic loss of cardiomyocytes of the heart is a main contributor for the poor cardiac function that culminates in heart failure [2]. But the adult myocardium has very limited potential of self-regeneration [3]. Therefore, myocardium protection and regeneration are considered as important means of heart disease treatment. Stem cell transplantation has become a new strategy for the treatment of myocardial infarction (MI) and heart failure [4].

Mesenchymal stem cells (MSCs) seem to be highly advantageous for cellular therapy since they are safe, multipotent, and immune-privileged [5]. Adipose-derived mesenchymal stem cell (ADSC) emerged as a preferable cell source to treat MI, due to its advantages over other MSC types: easy accessibility, minimal morbidity upon harvest, clinically relevant abundance without expansion, stronger proliferative capacity [6], [7].

Growing evidence indicates that ADSC transplantation improves cardiac function of post-MI heart via direct differentiation and paracrine effect [8], [9]. Ideally, new myocardium can be build by direct differentiation of ADSC into cardiac cells [10], [11], and ADSC paracrine factors can induce cytoprotection and angiogenesis [12]. However, 1) the evidence to support the therapeutic role of paracrine mechanism in animal model of MI is lacking; 2) The capacity of ADSC differentiation into cardiomyocytes and vascular cells in infarcted hearts has been controversial; 3) The relative contribution of paracrine effect versus direct differentiation in the ADSC mediated beneficial effect is unclear.

Our study was designed to evaluate the capacity of ADSC conditioned medium in the prevention of heart failure, and compare its therapeutic effect with ADSC transplantation in the MI mice. We also defined the differentiation capacity of ADSC towards cardiac and vascular lineages, and further assessed the relative contribution of paracrine effect versus direct differentiation in ADSC induced cardioprotection.

## Materials and Methods

### 1. Human adipose-derived stem cell (ADSC) culture and lentiviral transfection of green fluorescent protein (GFP)

Human ADSCs were isolated by using a modified protocol, as described previously [8]. Human abdominal subcutaneous adipose tissue was obtained from 11 healthy adults (20–30 year-old, female) during liposuction. The tissue was obtained per a tissue acquisition protocol approved by the Medical Ethics Committee of the Third Military Medical University (Permit Number: 2012[13]). All the tissue donors involved have provided written informed consent, and the consent procedure was developed and approved by the Medical Ethics Committee. Adipose tissue was minced and incubated for 45 minutes at 37°C on a shaker with 0.1% collagenase I (Worthington) in phosphate-buffered saline (PBS) with 2 mM calcium chloride. The digested tissue was sequentially filtered through 100 µm and 40 µm filters (BD) and centrifuged at 300 g for 5 minutes at room temperature. The supernatant containing adipocytes and debris was discarded.

ADSCs were re-suspended in Dulbecco's modified eagle medium (DMEM)/F12 (Gibco) containing 10% fetal bovine serum (FBS), 2 mM glutamine, 100 U/ml penicillin, 100 µg/ml streptomycin and then cultured at 37°C with 5% CO_2_. ADSCs of passage 3 were used for experiments.

To track the ADSCs after transplantation in heart tissue, ADSCs were genetically labeled with green fluorescent protein (GFP). Lentiviral vector encoding GFP gene (purchased from Genechem Co., Ltd., Shanghai, China) was used with an MOI of 10∶1. An infection cocktail containing lentiviral supernatant, 5 mg/mL polybrene, and 10% FBS in DMEM/F12 with 2.5×10^5^ target ADSCs were cultured in 60 mm dishes for 24 hours. To enrich the genetically modified ADSCs, the GFP+ ADSCs were sorted with FACSAria II (BD Biosciences) after the lentiviral transfection. The purified GFP^+^ ADSCs (>99% purity) were then expanded in culture for use.

### 2. Flow cytometry

To analyze the cell surface phenotype, ADSCs at passage 3 were fixed for 10 minutes in 1% paraformaldehyde. Cells were then washed twice with PBS and stained with primary antibodies at room temperature for 30 minutes. The primary antibodies include anti-human CD34-PE, CD90-FITC, CD73-PE, CD44-FITC, CD105-PE and CD45-FITC. Isotype-matched mouse IgGs were used as controls. Flow cytometric analysis was performed on a fluorescence-activated cell sorter (BD FACSCalibur). Data analysis was performed with Flowjo software.

### 3. Adipogenesis and osteogenesis

The multi-lineage differentiation potential of ADSCs before and after lentiviral-GFP transfection was investigated as described previously [8]. Briefly, lipogenic differentiation was assessed by incubating ADSCs for 3 weeks in adipogenic medium containing low-glucose DMEM (Gibco) supplemented with 10% FBS, 100 µM L-ascorbate acid, 1 µM dexamethasone, 0.5 mM 1-methyl-3-isobutylxanthine, 100 µM indomethacin, and 10 µg/ml human recombinant insulin. Cells from the control group were cultured in low-glucose DMEM plus 10% FBS (control medium). Adipogenesis was assessed by incubating cells with Oil Red O solution to stain neutral lipids in the cytoplasm. Osteogenic differentiation was performed by incubating ADSCs for 3 weeks in osteogenic medium containing high-glucose DMEM supplemented with 10% FBS, 0.1 µM dexamethasone, 200 µM L-ascorbic acid, and 10 mM β-glycerol phosphate. Cells in the control group were cultured in high-glucose DMEM plus 10% FBS. To assess mineralization, calcium deposits in cultures were stained with Alizarin Red S and then observed under a phase-contrast microscope. All the media were changed every 3 days. All the materials and reagents were from Sigma-Aldrich unless indicated otherwise.

### 4. Preparation of conditioned medium (CM)

To prepare the CM of ADSCs, we used a modified method from that described previously [13], [14]. 80% confluent GFP-ADSCs were washed with PBS and then fed with serum-free medium for 24 hours. The medium was then collected and used for *in vitro* experiments. For *in vivo* experiments, the CM was collected and the cells were counted for normalization purpose. For each animal, we used CM generated by 1×10^5^ ADSCs. The CM was centrifuged at 300 g for 5 minutes, and sterilized through a 220 nm filter. The collected CM was concentrated using Amicon Ultra-15 centrifugal filter units (5 kDa cut-off; Millipore). Control medium (termed DMEM for convenience) was generated in the same way except there were no cells in the plate.

### 5. Isolation and culture of neonatal rat ventricular myocytes (NRVMs)

Primary cultures of NRVMs from 1-2-day-old Sprague-Dawley rat pups were performed using the method described in detail previously [15], [16]. Immediately after euthanasia of the pups, hearts were removed, ventricles were minced, and heart cells were dissociated with trypsin (1.5 mg/mL, Difco). Dissociated cells were then collected at 10-minute intervals. The cells were re-suspended in DMEM supplemented with 10% FBS and 0.1 mM BrdU, and plated in culture dishes for 60 minutes to allow fast-adherent fibroblasts to attach. Non-adherent cells (NRVMs) were collected and plated in other culture dishes or coverslips coated with fibronectin at a density of 160 cells/mm^2^. The following day, the medium was replaced with fresh medium without BrdU.

Experiments using the animals were conducted with the approval of the Animal Care and Use Committee of Third Military Medical University (Approval ID: SCXK (Military) 2007015), according to the State Science and Technology Commission Regulations for the Administration of Affairs Concerning Experimental Animals (1988, China). All surgery was performed under isoflurane anesthesia, and all efforts were made to minimize suffering.

### 6. Mouse myocardial infarction (MI) and intramyocardial injection of DMEM, ADSC and ADSC-CM

Permanent ligation of the left anterior descending (LAD) coronary artery and cell injection were performed in 3–4 months old male severe combined immunodeficient mice or C57BL/6 mice as described previously [17]. Anesthesia was maintained with isoflurane inhalation. A permanent ligation was performed around the LAD coronary artery 2–3 mm from its origin with a 7-0 silk suture. Sham-operated animals were subjected to the same surgical procedures except that the suture was passed under the LAD but not tied.

Immediately after MI injury, donor GFP-ADSCs suspended in 50 µL DMEM were injected at 5 sites (10 µL per each site) in the anterior and posterior infarct border zones of the post-MI heart. For MI+DMEM and MI+ADSC-CM groups, the same volume of DMEM and concentrated ADSC-CM were intramyocardially injected at the same sites of the infarct border zones. For C57BL/6 mice, immune suppressant drug FK506 (3 mg/kg/day, i.p., Sigma) was administrated to achieve an equivalence to allograft transplantation throughout the duration of the study.

Animal care and all experimental procedures were performed in strict accordance with the approved protocols and animal welfare regulations of the Animal Care and Use Committee of the Third Military Medical University.

### 7. Area at risk (AAR) and infarct size determination

AAR was defined by Evans blue staining, and the infarct size was defined by triphenyltetrazolium chloride (TTC) straining. Briefly, 0.3 ml of 1.5% Evan's blue dye in PBS was injected retrograde into the brachiocephalic artery. The non-ischemic area, which was not at risk, was stained blue. The heart was excised and cut into five 1-mm-thick transverse slices, parallel to the atrioventricular groove. AAR was calculated by dividing the total non-blue area by the total area of the left ventricle (LV) sections. Each slice was incubated in a 1% solution of TTC at 37°C for 15 minutes to differentiate infarct area (pale) from viable (brick red) myocardial area. The ratio of the length of the infarct band to the total length the LV was calculated and expressed as a percentage of infarct size. The AAR and infarct size from each section were measured using Image J software, and the values obtained were averaged. The individuals conducting the experiment were blinded as to the experimental groups [18].

### 8. Echocardiography analysis

Echocardiography was performed (GE vivid 7 dimension) to determine cardiac structure and function in conscious mice [19], [20]. Hearts were viewed in the long and short axis between the two papillary muscles and each measurement was obtained with M-mode by averaging results from three consecutive heart beats. Parameters including diastolic left ventricle internal diameter (LVIDd) and systolic left ventricle internal diameter (LVIDs) were measured to determine structure changes in cardiac morphology. Percent fractional shortening (FS) was calculated as follows: %FS  =  (LVIDd – LVIDs)/LVIDd×100%. Left ventricular ejection fraction (EF) was automatically calculated by echocardiography according to the Teicholz formula. The individuals conducting the experiment were blinded as to the animal treatments.

### 9. Histopathology

Formalin fixed hearts were processed, embedded in paraffin and cut as 5 µm sections. To track the differentiation phenotypes of GFP^+^ ADSCs in hearts, anti-GFP antibody (Invitrogen), anti-von Willebrand factor (vWF) antibody (Santa Cruz), anti-smooth muscle actin (SMA) antibody (Santa Cruz) anti-sarcomeric tropomyosin antibody (Sigma) and anti-α-actinin (Sigma) were used. To detect capillary density in peri-infarct myocardium, a rat anti-mouse CD31 was used [21]. To detect the cardiomyocyte apoptosis, an in situ apoptotic cell death detection kit (POD; Roche Applied Bio Sciences) based on the terminal deoxynucleotidyl transferase dUTP nick end labeling (TUNEL) assay was used [18]. The percentage of apoptotic nuclei per section was calculated by counting the number of TUNEL-staining nuclei divided by the total number of DAPI-positive nuclei. Cardiomyocyte cytoplasm was identified by anti-sarcomeric tropomyosin antibody. The number of capillaries and apoptosis was counted in 5 random fields per section of the peri-infarct zone, and a total of 5 random sections per animal were analyzed.

Alexa Fluor 488 or Alexa Fluor 647-conjugated secondary antibodies (Jackson ImmunoResearch) were applied appropriately. DAPI was used for nuclear counterstaining. Images were taken at a magnification of 20× with a Nikon fluorescence microscope (Tokyo, Japan). The manual counting was performed in a blinded fashion.

### 10. Statistical Analysis

Data are reported as means±SEM. For analysis of differences between two groups, student's t test was performed. For multiple groups, ANOVA was carried out followed by Student–Newman–Keuls test. A *p* value of <0.05 was considered significant. In this study, n is the number of cells examined, and N is the animal number or times of cell cultures.

## Results

### 1. The culture and characterization of ADSCs

**Figure 1:**
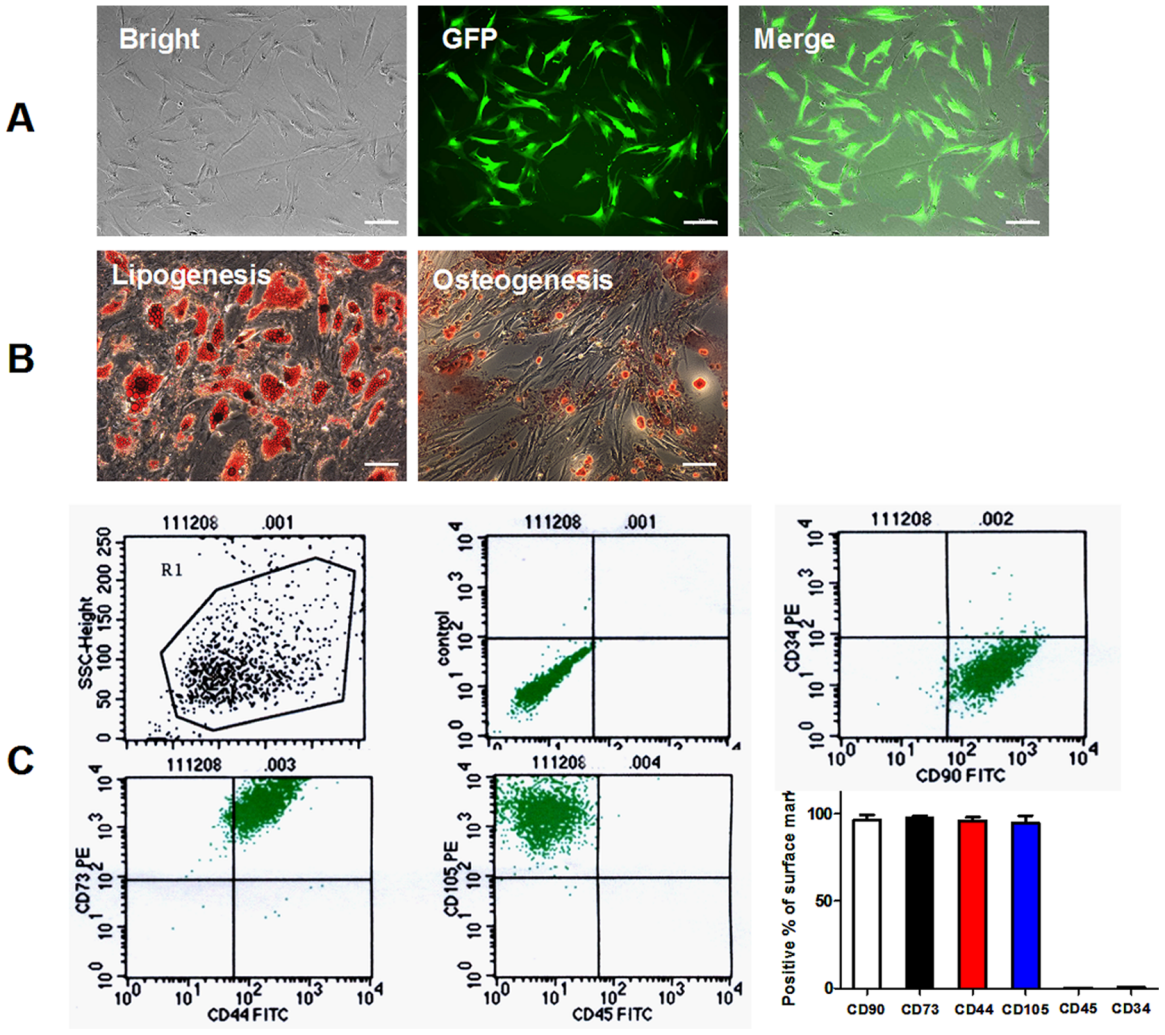
Characterization of human ADSCs and GFP labeling. A: Representative images of ADSCs transfected with lentiviral GFP. ADSCs showed fibroblast-like morphology and genetically labeled with GFP. B: Lipogenenic and Osteogenic differentiation of ADSCs lentivirally labeled with GFP. Histochemical staining of adipocytes (oil red O, left panel) and osteocytes (Alizarin red, right panel). C: Flow cytometric analysis shows that human ADSCs were positive for CD90, CD73, CD44 and CD105 and negative for CD34 and CD45. ADSC: adipose-derived mesenchymal stem cells. GFP: green fluorescent protein.

The primary cultured ADSCs of 3-5 passages were identified as to cell morphology, surface markers and multipotent differentiation potential. As shown in **Figure 1A**, these ADSCs showed a fibroblast-like cell morphology. Meanwhile, ADSCs transfected with lentiviral GFP showed green fluorescence. Similar to the previous study [8], ADSCs lentivirally labelled with GFP maintained the potential to differentiate into adipocytes and osteoblasts, demonstrating their multi-lineage differentiation potential. As shown in **Figure 1B**, ADSCs treated with lipogenic medium for 3 weeks were stained with oil red O to mark neutral lipids in the cytoplasm (left panel); ADSCs treated with osteogenic medium for 3 weeks were stained with Alizarin red to mark mineralized matrix (right panel). As shown in **Figure 1C**, flow cytometry demonstrated that these cells were positive for stem cell markers CD90^+^ (96.43±2.92%), CD73^+^ (97.12±1.17%), CD44^+^ (96.07±2.44%), CD105^+^ (94.74±4.23%), but negative for haematopoietic lineage markers CD45^−^ (0.15±0.10%) and CD34^−^ (0.96±0.12%). These results indicated that these cells maintain a typical ADSC phenotype, consistent with previous reports [22].

### 2. Survival rates of post-MI mice

**Figure 2:**
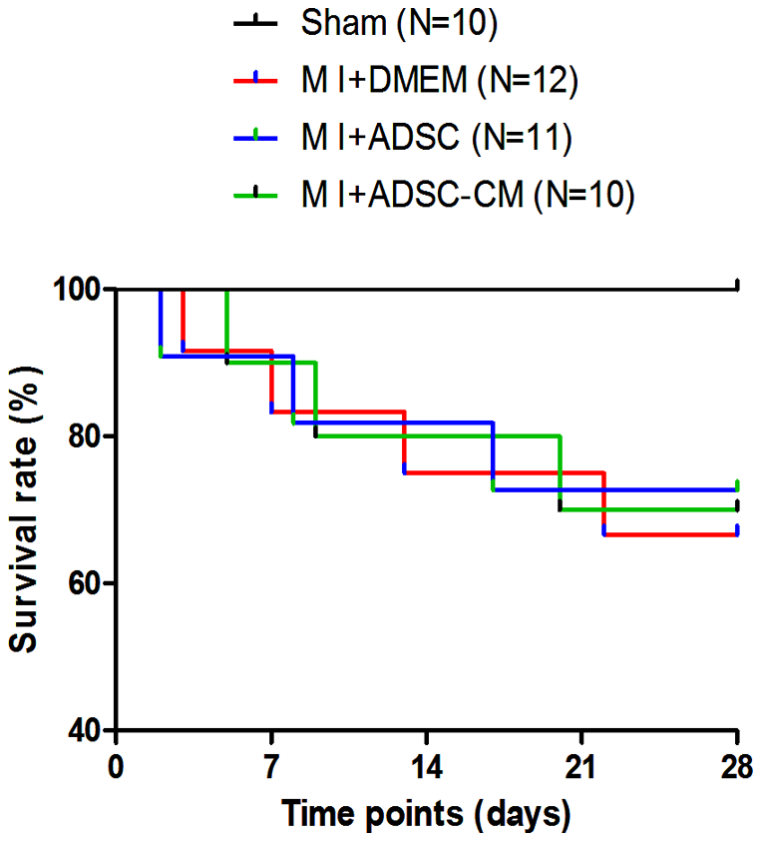
The survival rates of MI mice after treatment with ADSC or ADSC-CM. All the MI mice treated with DMEM, ADSC and ADSC-CM were observed up to 4 weeks. MI: myocardial infarction.

Coronary artery ligation or sham surgery was performed on mice. Our preliminary study showed that the rates of death during surgery were not different among the three groups (MI+DMEM: 14.3%; MI+ADSC: 16.7%; MI+ADSC-CM: 15.4%, respectively) before treatment. After MI injury, DMEM (control), ADSC and ADSC-CM were immediately injected into the infarct border zone. The mouse survival after treatments was observed up to 4 weeks. As shown in **Figure 2**, the survival rate of all groups with MI were significantly increased compared with sham-treated mice. However, neither ADSC nor ADSC-CM treatment reduced the survival rate compared to the MI-controls (MI+DMEM: 75.0%, N = 12; MI+ADSC: 70.0%, N = 10; MI+ADSC-CM: 72.7%, N = 11. p>0.05).

### 3. Infarct size and cardiac function of mice after MI

**Figure 3:**
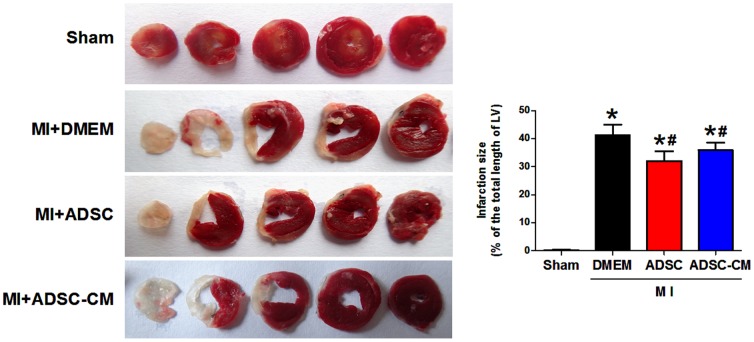
Infarct size in MI mice after treatment with ADSC or ADSC-CM. A: Representative images of TTC-stained heart sections obtained from Sham, MI+DMEM, MI+ADSC, and MI+ADSC-CM groups at 4 weeks post-transplantation. B, Graphic representation of the infarct size expressed as the ratio of the length of the infarct band to the total length of left ventricle (N = 7, *p<0.05 versus MI+DMEM; # p<0.05 versus MI+ADSC). TTC: triphenyltetrazolium chloride.

To compare the therapeutic potential of ADSC and ADSC-CM, we evaluated the effects of both therapies on infarct size and cardiac function in the setting of MI. Infarct area in heart tissue sections was measured with TTC staining. As shown in **Figure 3**, both ADSC and ADSC-CM treatment significantly reduced the infarct size at 4 weeks post-MI compared with control MI hearts (MI+DMEM: 41.9±3.0%; MI+ADSC: 32.2±2.8%; MI+ADSC-CM: 35.7±2.5%; p<0.05). However, the difference between the groups of ADSC and ADSC-CM is not statistically significant.

**Figure 4:**
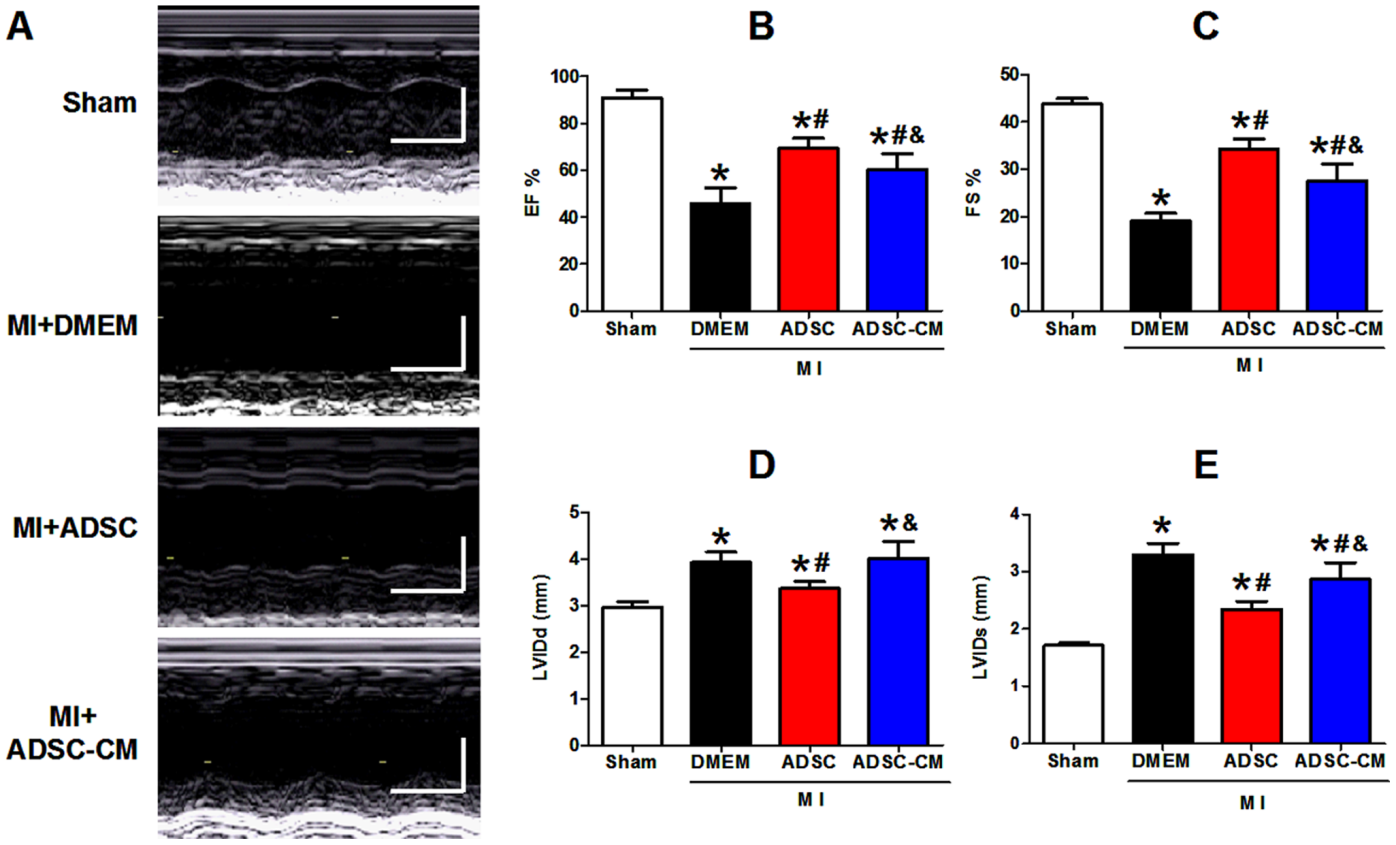
Cardiac function in MI mice after treatment with ADSC and ADSC-CM. A: Representative M-mode images (long-axis view) of hearts with sham surgery and infracted hearts 4 weeks post-MI. B-C: Left ventricular ejection fraction (EF) and fraction shortening (FS) at 4 weeks post-MI. D-E: Diastole left ventricle internal diameter (LVIDd, mm) and systolic left ventricle internal diameter (LVIDs, mm) at 4 weeks post-MI (N = 7, * p<0.05 versus Sham; # p<0.05 versus MI+DMEM; & p<0.05 versus MI+ADSC).

Left ventricle (LV) function and chamber dimensions of sham or infracted mice were examined 4 weeks post-MI using echocardiography. Representative M-mode images of hearts of different groups are shown in **Figure 4A**. In sham animals, LV contractile function was stable throughout the duration of this study. After MI, all animals had reduced pump function. As shown in **Figures 4B**–**C**, LV fractional shortening (FS) and ejection fraction (EF) were significantly decreased after MI in control mice (pre-MI versus post-MI: FS: 43.8±3.7% versus 19.0±3.8%; EF, 90.8±5.4% versus 45.6±6.8%, p<0.05). With ADSC-CM injection, FS and EF were significantly increased compared with control MI hearts (FS: 27.6±3.5%; EF: 60.4±5.8%, p<0.05 versus MI+DMEM). ADSC transplantation further enhanced the LV function compared with ADSC-CM treatment (FS: 34.3±2.5%; EF: 69.2±4.3%, p<0.05 versus MI+ADSC-CM). Besides, all hearts showed evidence of enlargement in the LV chamber dimensions (dilation) after MI. As shown in **Figures 4D**–**E**, diastolic left ventricle internal diameter (LVIDd) was increased from 2.97±0.11 mm at pre-MI to 3.93±0.22 mm of 4 weeks post-MI; systolic left ventricle internal diameter (LVIDs) was increased from 1.71±0.04 mm at pre-MI to 3.30±0.20 mm of 4 weeks post-MI (P <0.05). ADSC-CM did not change LVIDd (4.02±0.36 mm, P >0.05 versus MI+DMEM) but decreased LVIDs (2.87±0.29 mm, p<0.05 versus MI+DMEM). ADSC transplantation significantly reduced both LVIDd and LVIDs (LVIDd, 3.37±0.15 mm, p<0.05 versus MI+DMEM; LVIDs: 2.23±0.15 mm, p<0.05 versus MI+DMEM). These results indicate that ADSC-CM reduced the post-MI deterioration of cardiac function and structure, although these cardioprotective effects were less potent than those from ADSC transplantation.

### 4. The differentiation of transplanted ADSCs in post-MI heart

**Figure 5:**
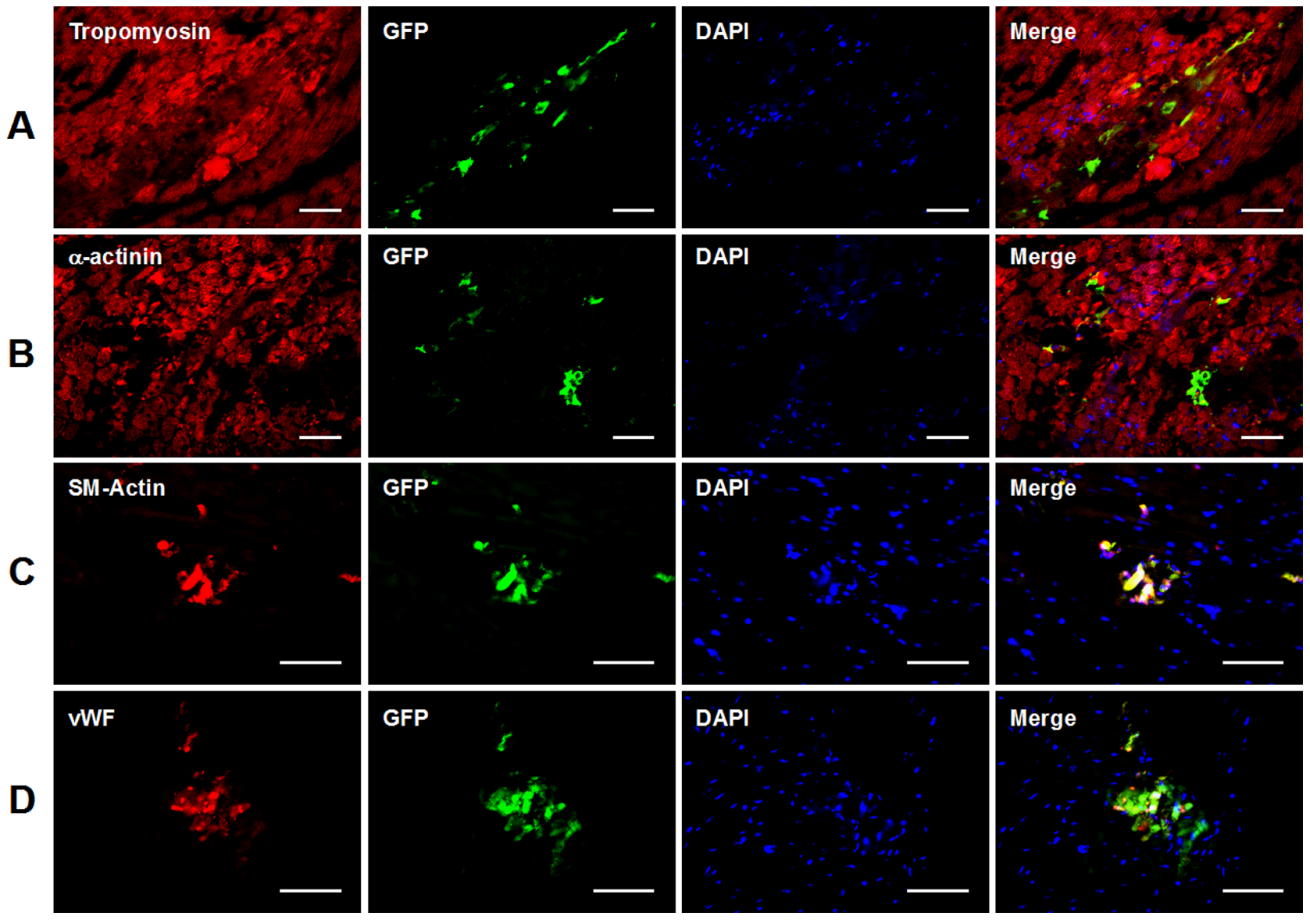
Immunofluorescence analysis of ADSC differentiation after transplantation in post-MI heart. A-B: Sections of hearts were triple-stained with DAPI (nuclei), antibodies to GFP and cardiomyocyte markers tropomyosin (A) or α-actinin (B) 4 weeks post-transplantation. C: Sections of hearts were triple-stained with DAPI, antibodies to GFP and smooth muscle cell marker smooth muscle actin (SMA) 4 weeks post-transplantation. D: Sections of hearts were triple-stained with DAPI, antibodies to GFP and endothelial cell marker von-Willebrand Factor (vWF) 4 weeks post-transplantation. Scale bars represent 50 µm. DAPI: 4′,6-diamidino-2-phenylindole.

Transplanted ADSCs labeled with lentiviral GFP were tracked by using antibody against GFP. As shown in **Figure 5**, clusters of injected GFP^+^ ADSCs were detected in hearts 4 weeks after cell transplantation, suggesting successful engraftment and survival of the injected ADSCs.

As mentioned above, ADSC and ADSC-CM treatments reduced infarct size, indicating a higher percentage of viable myocardium. To determine whether it resulted from myocardial regeneration, the cardiomyogenic events of transplanted ADSCs were examined by double staining with GFP and cardiomyocyte markers tropomyosin or α-actinin. As shown in **Figures 5A**–**B**, no GFP-positive ADSC-derivatives were found to express tropomyosin or α-actinin in all 7 hearts 4 weeks post-transplantation. This fact indicates that myocardial differentiation of ADSCs was not detectable, thus the reduced infarct size is not due to newly-formed myocardium differentiated from ADSCs. As shown in **Figures 5C**–**D**, ADSCs were demonstrated to express vWF or SMA, indicating that some injected cells differentiated into endothelial cells and smooth muscle cells.

Our observation also demonstrated an absence of GFP^+^ cell in mice treated with ADSC-CM, indicating no contamination of viral particles in the conditioned medium.

### 5. The contribution of ADSC direct differentiation to the enhanced angiogenesis in post-MI heart

**Figure 6:**
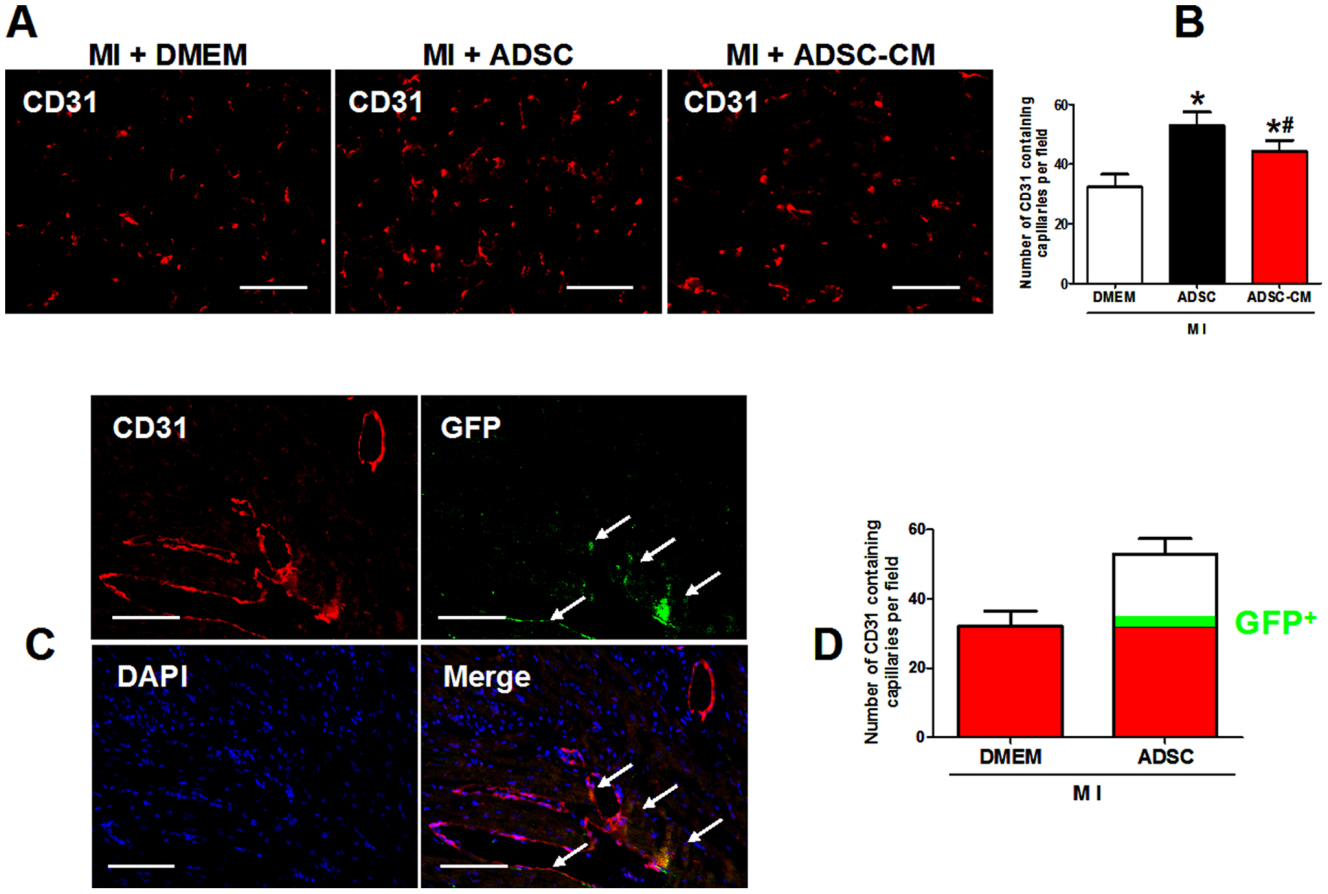
The contribution of ADSC differentiation to angiogenesis in post-MI heart. A-B: Representative figures and quantification of capillary density in infarct border zone at 4 weeks after ADSC transplantation analyzed by CD31 (endothelial cell marker) staining. *p<0.05 versus MI+DMEM; # p<0.05 versus MI+ADSC (N = 7). C: Representative figures of immunostaining GFP, CD31 and DAPI in the infarted hearts 4 weeks after ADSC transplantation. Arrows indicate GFP^+^/CD31^+^ capillaries, suggesting newly formed capillaries derived from transplanted ADSCs. Scale bars represent 50 µm. D: Quantification of the number of ADSC derived capillaries relative to overall increases in capillary density. In the diagram, GFP^+^ capillaries take about 9% of the increased capillary density compared to control (MI+DMEM). N = 6.

As shown in **Figures 6A-B**, we demonstrated that treatment with ADSC-CM induced a significant increase in capillary density in infarct border zone 4 weeks post-MIThis *in vivo* data further confirmed the previous finding that ADSC-CM promotes endothelial cell survival and migration *in vitro* [23]. Moreover, our data showed that the proangiogenic effect of ADSC was superior to ADSC-CM treatment (capillaries per field stained by CD31: MI+DMEM: 32.2±4.3; MI+ADSC 52.9±4.5; MI+ADSC-CM: 44.4±3.6; p<0.05).

As a means of quantifying the degree of improvement on angiogenesis which is attributed to direct regeneration versus indirect paracrine effects, we calculated the relative contribution of ADSC-differentiated vascular cells to the capillary density in the infarcted hearts 4 weeks after ADSC transplantation. The double staining of GFP and CD31 showed GFP^+^ capillaries **(** **Figure 6C** **)**, indicating that the endothelial cells differentiated from ADSCs were involved in angiogenesis. By calculation, about 3.4±0.7% of the total capillaries were found to be of human ADSC origin. Because of an overall 64.3% increase of capillary density in ADSC treated mice compared to control MI mice treated with DMEM, ADSC differentiation amounted to about 9% of the enhanced angiogenesis (**Figure 6D**). Presumably, the other 91% of the ADSC transplantation induced angiogenesis was mediated by paracrine effect. Therefore, paracrine effect predominate the mechanisms for ADSC induced angiogenesis, although ADSC direct differentiation played an unambiguous role.

### 6. The effect of ADSC and ADSC-CM on cardiomyocyte apoptosis in infarct border zone

**Figure 7:**
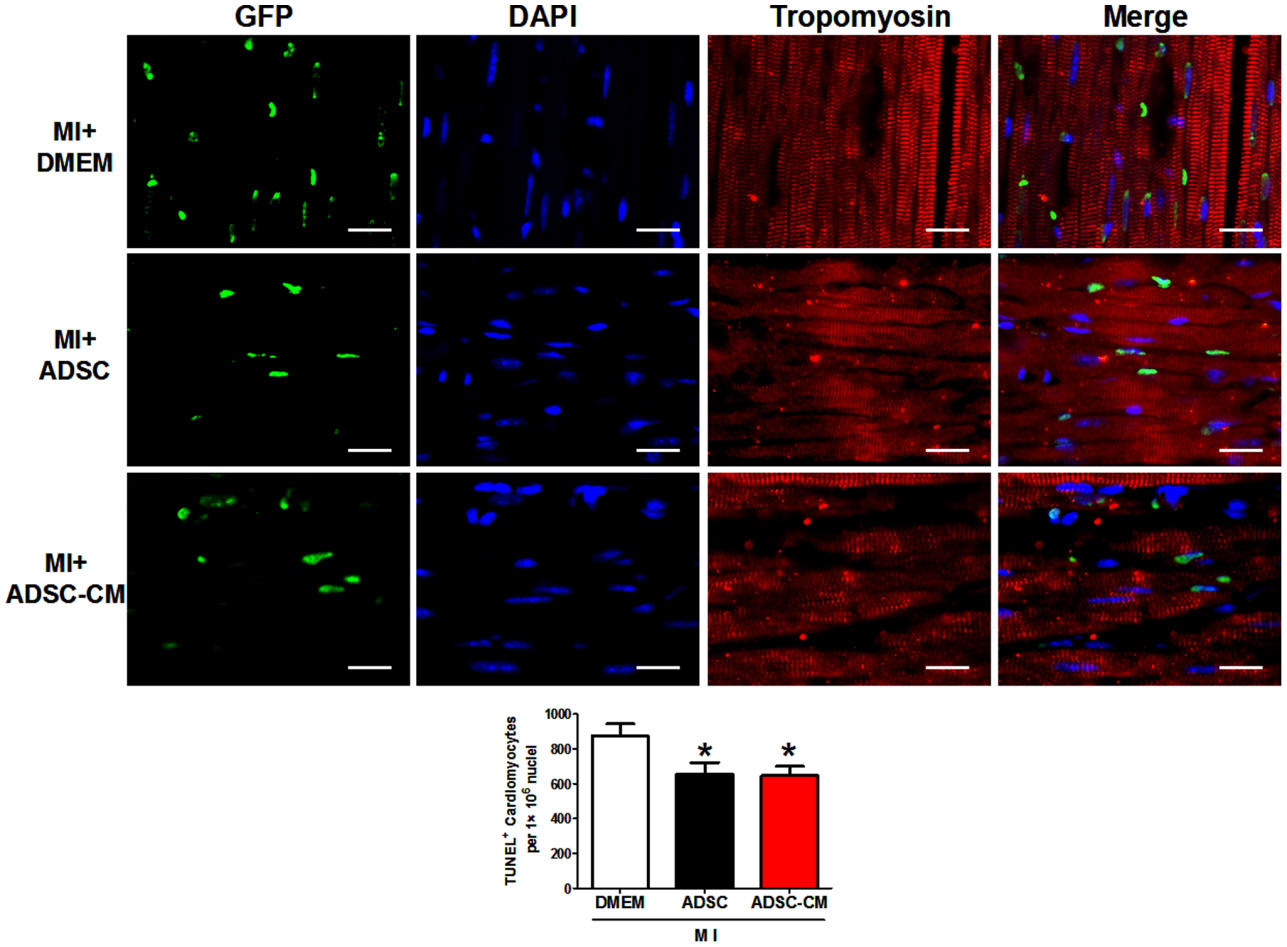
The effect of ADSC and ADSC-CM on cardiomyocyte apoptosis in post-MI heart. Representative figures and quantification of TUNEL positive cardiomyocytes in infarct border zone at 2 days after treatment of DMEM, ADSC or ADSC-CM. TUNEL positive nuclei are stained green, tropomyosin is red and DAPI is blue. * p<0.05 versus MI+DMEM, N = 6. Scale bars represent 30 µm.

Cardiomyocyte apoptosis is a main contributor for the progression of acute MI [2], [24]. To determine the effects of ADSC and ADSC-CM treatment on cardiomyocyte apoptosis, we used TUNEL assay to measure apoptotic cell death in the infarct border zone 2 days after MI. As shown in **Figure 7**, ADSC transplantation significantly reduced TUNEL positive cardiomyocytes (number per 10^6^ nuclei: Control: 867.3±67.9; ADSC: 657.4±64.2; p<0.05). Interestingly, ADSC-CM exerted a similar anti-apoptotic effect on cardiomyocytes (ADSC-CM: 646.7±54.1, p>0.05 versus ADSC), suggesting both treatments.

### 7. The effect of ADSC-CM on cardiomyocyte apoptosis in vitro

To verify the cardioprotective effect of ADSC-CM, we used ADSC-CM to pretreat NRVM subjected to oxidative stress. Administration of 100 µM H_2_O_2_ for 6 hours was used to induce oxidative injury. NRVM apoptosis was detected by western blot analysis of the ratio of cleaved caspase-3/total caspase-3 and TUNEL staining analysis.

**Figure 8:**
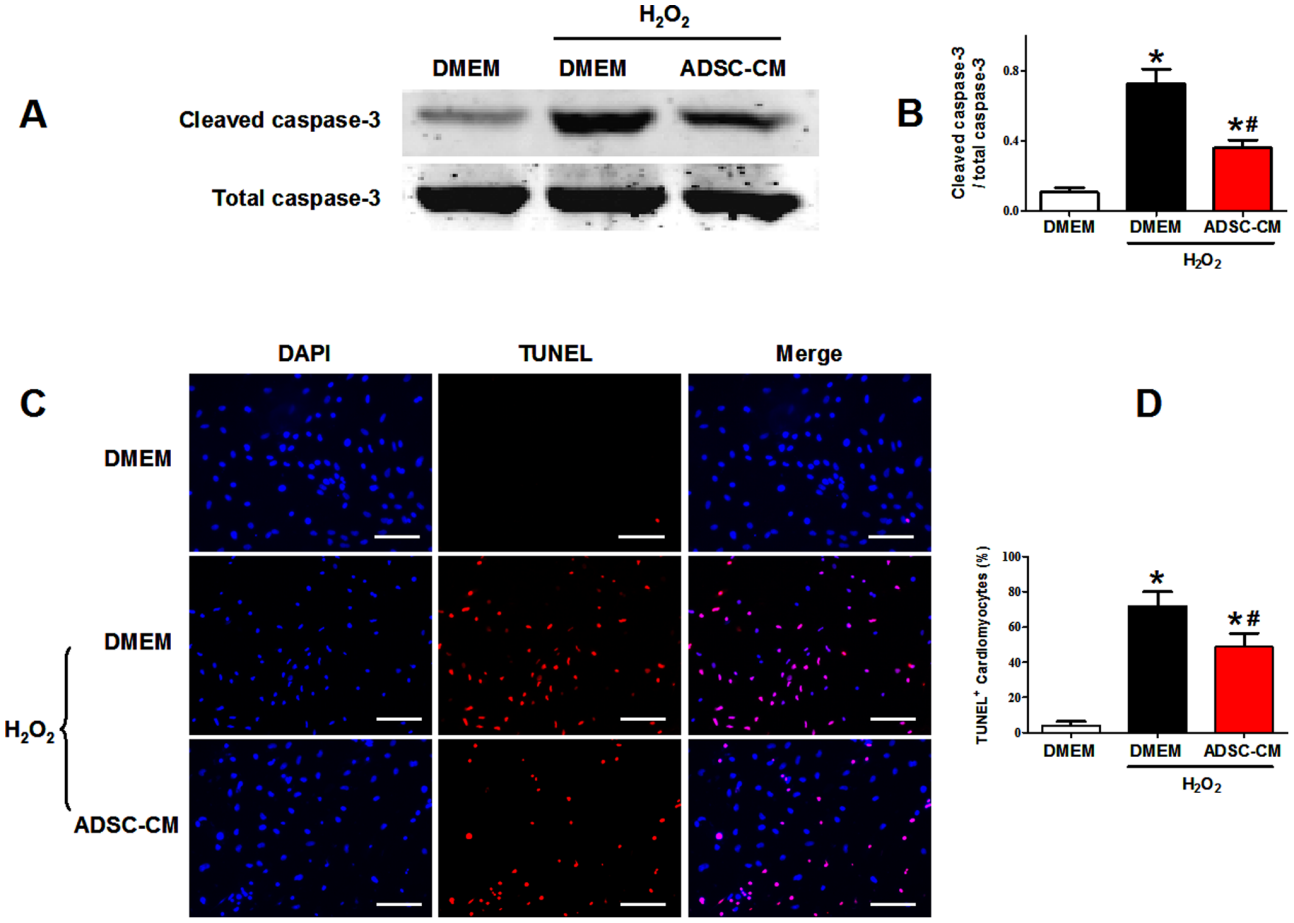
The antiapoptotic effect of ADSC-CM on cardiomyocytes subjected to oxidative stress. A-B: Representative figures (A) and quantification (B) of western blotting analysis of cleaved caspase-3 of NRVMs subjected to H_2_O_2_ treatment (N = 5). C: Representative images of TUNEL labeling (TMR-red) and cell nuclei (DAPI-blue) of cardiomyocytes under H_2_O_2_ treatment. D: Quantification of TUNEL staining. TUNEL positive rate = (TUNEL-positive nuclei/DAPI-positive nuclei)×100% (N = 9). * p<0.05 versus control without H_2_O_2_; # p<0.05 versus control with H_2_O_2_. Scale bars represent 100 µm.

As shown in **Figures 8A-B**, the cleaved caspase-3 protein was significantly increased in NRVMs subjected to H_2_O_2_ injury (relative intensity to total caspase-3: control: 0.107±0.026; H_2_O_2_: 0.724±0.087; p<0.05); Pretreatment of ADSC-CM reduced cleaved caspase-3 expression compared to H_2_O_2_ group (relative intensity to total caspase-3: H_2_O_2_+ADSC-CM: 0.362±0.043, p<0.05 versus H_2_O_2_). As shown in **Figures 8C-D, **H_2_O_2_ treatment increased TUNEL-positive NRVM compared to control (TUNEL-positive rate: control: (4.18±2.11)%; H_2_O_2_: (71.65±8.31)%; p<0.05). Pretreatment of ADSC-CM significantly reduced the TUNEL-positive rate (H_2_O_2_+ADSC-CM: (48.64±7.70)%, p<0.05 versus H_2_O_2_). These results showed that ADSC-CM significantly reduced H_2_O_2_-induced cardiomyoyte apoptosis, indicating the ADSC paracrine protective effect on the myocardium.

## Discussion

In the present study, we demonstrated that ADSC-CM treatment is sufficient to improve cardiac function post-MI mice, through reducing cardiomyoycte apoptosis and enhancing angiogenesis. ADSC transplantation exerts a moderately superior therapeutic potential to ADSC-CM injection. Transplanted ADSC is able to differentiate into endothelial cells and smooth muscle cells, which contributes to about 9% of the ADSC mediated angiogenesis. Thus, ADSC induced beneficial effect is predominantly mediated by paracrine mechanism, of which direct differentiation plays a minor role.

As a suitable cell type for treating heart disease [25], ADSC transplantation improves cardiac function in the post-MI hearts [8]. The mechanisms underlying the beneficial effect of ADSCs on cardiac injury are not fully understood, although it is known that its two major actions include direct differentiation and paracrine effect.

Recent studies showed that conditioned medium (CM) of human ADSCs exerts therapeutic effects on murine ischemic stroke models, mainly by increasing endothelial cell proliferation and reducing neural cell apoptosis [26], [27]. However, whether CM of human ADSCs is sufficient to treat MI injury in animal models is unknown. We, for the first time, demonstrated that intramyocardial injection of ADSC-CM is able to reduce infarct size and improve cardiac function in infarcted mice. We further demonstrated that ADSC-CM promotes cardiomyocyte survival and angiogenesis *in vivo,* which echoes a previous *in vitro* finding that ADSC-CM promotes endothelial cell survival and migration [23]. Our data thus provide direct evidence for the therapeutic potential of ADSC paracrine action in animal MI models.

The differentiation potential of transplanted ADSCs in the ischemic heart by transforming into the cardiac lineages has been controversial. Some reports show that ADSC possesses a capacity to transdifferentiate to cardiomyocyte, endothelial cell and smooth muscle cell after transplantation into the heart [7], [28]. Other reports show an opposite finding of no specific differentiation of human ADSC to those cell lineages [29]. In the present study, we demonstrated that some surviving ADSCs differentiated into endothelial and smooth muscle cells after transplantation. These ADSC-differentiated vascular cells contributed to angiogenesis and were incorporated in the heart vasculature, which confirmed the plasticity of ADSCs toward endothelial cells [30] and ADSCs induced angiogenesis [6], [31]. On the other hand, no newly-formed cardiomyocyte derived from ADSC was found in our experiments, which disagrees with a recent *in vivo* study [32] but is in agreement with the finding that ADSC do not undergo cardiomyogenesis in an *in vitro* system co-cultured with NRVMs [33]. We only used markers tropomyosin and α-actinin for identifying new cardiomyocytes, which is a limitation of the present study and might account for the discrepancy between our finding and some previous studies.

Moreover, the relative roles of direct differentiation versus paracrine mechanism in the use of ADSC treatment for cardiac repair are inconclusive. We carefully compared the therapeutic effect of ADSCs and CM collected from the same amount of ADSCs. As a result, ADSC transplantation induced a moderately stronger beneficial effect on infarct size and cardiac function than ADSC-CM injection. This might be ascribed to the stronger angiogenesis in ADSC treated infarcted hearts than ADSC-CM treated ones, giving the fact that both treatments exerted a similar anti-apoptotic effect on cardiomyocytes. Furthermore, ADSC direct differentiation into vascular cells, although significant, explains only 9% of the enhanced angiogenesis as compared to control, and about 20% of the surplus angiogenesis as compared to ADSC-CM. The discrepancy of proangiogenic effects between the two groups may be due to the fact that surviving ADSCs continue to secrete cytokines after transplantation but a single induction of ADSC-CM injection exerts only a transient effect. Besides, transplanted ADSCs are subjected to hypoxic stress, which increases the production of several of the stem cell paracine factors [12]. A previous study reported that direct differentiation contributes 20–50% of the beneficial effect of cardiac stem cell transplantation [34]. Our ratios are even lower than those of cardiac stem cells, which may be due to the different properties of the different stem cell types especially the stronger cardiomyogenic capacity of cardiac stem cells [10].

In the present experiment, each animal in ADSC-CM group was administrated with single injection of 50 µL concentrated ASDC-CM collected from 1×10^5^ ADSCs (20-fold concentrated), as the same injection volume and frequency of the ADSC transplantation. It is speculated that the beneficial effects of ADSC-CM can be optimized by using a higher concentration (100-fold for example [27]) and (or) increasing the injection frequency. Low survival rate, low differentiation efficacy and potential tumorigenicity of implanted cells could undermine the efficacy of the stem cell-based treatment [1], [35]. The strategy of using CM of stem cells may be a feasible approach to overcome these limitations [26], thus it is promising to translate stem cell-secreted paracrine products into better therapeutic strategies [36]. The paracrine products include cytokines/growth factors and recently reported microvesicles[36]. Various cytokines/growth factors, such as VEGF, HGF, IGF-1, sfrp2, can regulate cell apoptosis, angiogenesis, inflammation, and mobilization of endogenous cardiac stem cells [37], [38], [39]. Biologically active microvesicles, which carry RNA or microRNA, may inhibit cell apoptosis and target organs for repair purposes in various experimental models [36], [40].It is important to identify the specific factors involved in the ADSC-CM mediated cardiaoprotective effect. To use those identified critical factors among stem cell secretory products might be a meaningful treatment strategy for clinical applications.

On the other hand, the enhanced reconstruction in micro-vasculatures brought by transplanted ADSCs could be the alternatives of how cell therapy has a great promise to treat patients, despite the cell engraftment after transplantation was limited in long-term observation.

In conclusion, ADSC transplantation increases myocardial viability, enhances angiogenesis, ameliorates infarct size and improves cardiac function in MI mice. This therapeutic effect is predominantly mediated by a paracrine mechanism, while direct differentiation plays a minor role by contributing to angiogenesis.
